# Bateman's principles and human sex roles

**DOI:** 10.1016/j.tree.2009.02.005

**Published:** 2009-06

**Authors:** Gillian R. Brown, Kevin N. Laland, Monique Borgerhoff Mulder

**Affiliations:** 1 School of Psychology, University of St Andrews, South Street, St Andrews, Fife, KY16 9JP, UK; 2 School of Biology, University of St Andrews, Bute Medical Building, Queen's Terrace, St Andrews, Fife, KY16 9TS, UK; 3 Department of Anthropology, University of California, Davis, California 95616-8522, USA

## Abstract

In 1948, Angus J. Bateman reported a stronger relationship between mating and reproductive success in male fruit flies compared with females, and concluded that selection should universally favour ‘an undiscriminating eagerness in the males and a discriminating passivity in the females’ to obtain mates. The conventional view of promiscuous, undiscriminating males and coy, choosy females has also been applied to our own species. Here, we challenge the view that evolutionary theory prescribes stereotyped sex roles in human beings, firstly by reviewing Bateman's principles and recent sexual selection theory and, secondly, by examining data on mating behaviour and reproductive success in current and historic human populations. We argue that human mating strategies are unlikely to conform to a single universal pattern.

In *The Descent of Man*, Charles Darwin [1] noted that, throughout the animal kingdom, ‘the males of almost all animals have stronger passions than the females. Hence it is the males that fight together and sedulously display their charms before the female’ (Ref. [1], p. 272). Darwin erroneously suggested that eagerness of males ultimately resulted from the lower costs of transporting small sperm compared to the costs of moving relatively larger eggs [1]. The first compelling explanation of why competitiveness (see Glossary) and choosiness might differ between the sexes was provided by Bateman [2] in an experimental study of fruit flies (*Drosophila melanogaster*). Bateman's famous experiments showed that the number of offspring fathered by a male *Drosophila* increased with his number of mates, whereas a female fruit fly did not gain an increase in number of offspring from mating with several males. Bateman concluded that, because single ova are more costly to produce than are single sperm, the number of offspring produced by a female fruit fly was limited mainly by her ability to produce eggs, whereas the reproductive success of a male was limited by the number of females that he inseminated. He also stated that, in our own species, the sex difference in gamete size would result in greater within-sex competition amongst males than females [2].

The importance of Bateman's idea to evolutionary theory was brought to prominence by Robert Trivers [3], who drew attention to postzygotic parental investment, such as feeding young and defence against predators. Trivers predicted that the sex with the largest parental investment, usually female, would become a limiting resource for which members of the other sex compete. When females invest more than males, the ratio of reproductively available males to females (the operational sex ratio [OSR] [4]) is assumed to be male-biased. In these situations, reproductive success would be expected to vary more amongst males than females, with females competing less intensely for mates and seeking out fewer partners than males [3,5]. Apparently in support of this argument, greater variance in male than female reproductive success has been reported in some insects, frogs, lizards, birds and mammals [3,6]. Conversely, in sex-role-reversal species with high levels of paternal investment, females are predicted to compete more intensely than males for mates because males limit female reproductive success [7].

The aim of this paper is to review data on variance in reproductive and mating success and on the shape of the relationship between these variables in current and historic human populations, and to consider the implications of variation between populations for our understanding of human sex roles.

## Bateman's principles and sex-role evolution

Arnold [8] suggested that it is useful to recognize that Bateman actually derived three principles from his data on fruit flies: (i) males showed greater variance in number of offspring (reproductive success [RS]) than females; (ii) males showed greater variance in number of sexual partners (mating success [MS]) than females; and (iii) there was a stronger relationship between RS and MS among males than females (note, Bateman measured mating success as the number of partners with which offspring were produced; therefore, matings that failed to produce offspring were not included: see Ref. [9] for further critical evaluation of Bateman's experimental design and analyses). Here, we adopt Arnold's terminology and henceforth refer to Bateman's first, second and third principles.

Importantly, Bateman's third principle is key to predicting the potential for sexual selection to act on males and females. By itself, a sex difference in the variance of RS (first principle) or MS (second principle) provides no information about whether selection is predicted to act more strongly on males or females, because sex differences in variances can arise simply from random mating together with sex differences in handling times [10,11]. In addition, although variation in RS or MS is a precondition for selection to occur, sexual selection will only take place if the likelihood of success is dependent upon the possession of a particular trait. The slope of the regression line that relates RS to MS is known as the Bateman gradient (or sexual selection gradient), and whichever sex has the steepest gradient is likely to be the sex that experiences the strongest selection pressure on traits that enhance mating success [12,13].

Conventionally, male animals are assumed to be competitive and promiscuous, whereas females are assumed to be non-competitive and choosy. The term ‘sex role’ can be used to describe the behaviour patterns expected to be shown by males and females when competing for or choosing mates, although behavioural ecologists sometimes use the term more specifically to refer to the relative competitiveness of males and females for mates [14,15]. We use the broader meaning of the term sex role.

The pattern of sex roles within and across species will depend upon the relative shapes of the Bateman curves for each sex. Arnold [8] noted that researchers have too readily assumed that Bateman's observed relationships between RS and MS in *Drosophila* are universal, in terms of both their shapes and their sex-specificity. For instance, reviews of the insect literature have found little evidence either that male reproductive success increases invariably with number of matings, or that mate number is unimportant for females [16,17]. Even in fruit flies, there are reservations about the general applicability of Bateman's results [18,19]. Arnold [8] identified four possible relationships between RS and MS – linear, single-mate saturation, diminishing returns and intermediate optimum. Because empirical evidence can be found for all of these in both male and female animals (Box 1), it is clear that animals, including human beings, will not necessarily exhibit the original Bateman gradients as described for *Drosophila*.

Perhaps most incongruous with Bateman's original argument is the finding that females can benefit from multiple matings. Sarah Blaffer Hrdy was one of the first researchers to challenge the notion that female animals should be universally characterized as coy and choosy, based on her research on female primates [20]. Females can gain benefits, such as reduced infanticide risk or assurance of fertilization, from mating with multiple males (polyandry) [21,22]. Similarly, the assumption that males will always exhibit indiscriminate mating has also been challenged by comparative studies, particularly in insects, in which the energetic costs of sperm production, courtship and copulation can select for male choosiness and the prudent allocation of mating effort [23,24].

These variations in the data are reinforced by recent sexual selection theory, which has revealed greater complexity in the evolution of sex roles than hitherto conceived [25]. Mathematical models have shown, for example, that intense competition in one sex does not necessarily translate into choosiness in the other [11,26,27]. Individuals of either sex can both be discriminating in mate choice and can compete over access to mates. In addition, multiple factors have been predicted to affect the evolution of choosiness, competitiveness and parental care. Choosy individuals will increase their average quality of accepted mates at the cost of a reduced mating rate, whereas individuals that are not choosy will increase their mating rate but with less fitness gained per mating [27]. The trade-off between number and average quality of mating partners is predicted to be influenced by factors such as sex differences in the mortality costs of breeding, the costs of mate searching, rates of encountering potential mates and variation in mate quality [27], leading to the expectation that sex roles will vary within and between species (Box 2).

This theory leads to the following predictions about when it is likely that females will be choosy, when males will be choosy and when both or neither sex will be choosy: (i) females will be choosy in populations with a male-biased OSR, little paternal investment (which typically increases the cost of breeding to females), and/or considerable variation in male quality; (ii) males will be choosy in populations with a female-biased OSR, considerable paternal investment, and/or considerable variation in female quality; (iii) both sexes will be choosy when encounter rates are high, particularly where the parental investments of both sexes are large and not too different, and/or where variation in mate quality of both sexes is high; and (iv) neither sex will be choosy in less dense populations with low encounter rates and equal OSR.

We should not assume from these predictions that the sex showing greater choosiness will necessarily have lower variation in mating rate; for example, in a female-biased population, males might exhibit greater choosiness than females but, owing to a higher encounter rate with high-quality females, some males might be particularly successful.

Given that human populations are highly likely to vary in important measures that affect sex roles (such as adult sex ratios and population density), we might expect to see Bateman gradients differ between human populations. As in many non-human populations, a variety of alternative strategies are likely to characterize the mating (and/or marital) strategies of men and women, with each individual's optimal strategy dictated as much by the behaviour of same sex as opposite sex individuals; we would accordingly expect multiple strategies within each sex.

## Do human beings conform to Bateman's principles?

Here, we examine the available data for each of the three Bateman principles in human beings and examine which factors might explain variation across populations.

### Is there greater variation in RS among human males than females?

What we need to answer this question are comprehensive data on the variance in number of children produced by males and females across different populations. Such data are difficult to collate, not least because paternity is more difficult to ascertain with confidence than is maternity. Nonetheless, several datasets do exist (Table 1). When all populations of human beings are considered together, males exhibit higher mean variation in RS than females (one-sample *t* test, *t*_*17*_ = 3.82, p = 0.001). However, despite the expectation that male variance in RS will always exceed female variance [28], these data reveal large inter-population variation in the ratio of male to female variance in RS, ranging from 0.79 to 4.75. This variation between populations with regard to Bateman's first principle is inconsistent with the universal sex roles that Bateman envisaged [2].

**Figure 1:**
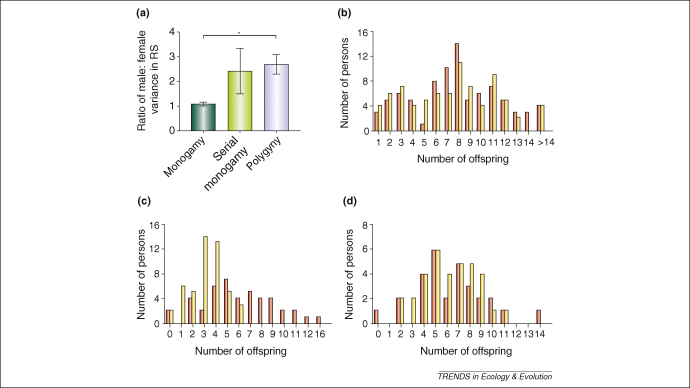
(a) The ratio of male to female variance in reproductive success varies significantly with mating system (Kruskal-Wallis, χ-squared = 9.09, df = 2, p = 0.011). Analyses were carried out on dataset from the monogamous (N = 6), serially monogamous (N = 3) and polygynous (N = 9) populations shown in Table 1. *Post hoc* analyses revealed that the ratio was significantly higher for polygynous populations than monogamous populations (*=p < 0.017 using Mann-Whitney U test). No other pairwise comparisons were significant. (b) Lifetime reproductive success of the monogamous Pitcairn Islanders, Pacific Ocean (re-drawn, with permission, from Ref. [82]). A Levene's test indicates that male and female variances are not significantly different (t = –0.15, n.s.). (N = 145 males and 127 females.) Individuals with zero offspring are not shown in the graph (N = 60 males and 47 females). (c) Lifetime reproductive success of the highly polygynous Dogon of Mali (re-drawn, with permission, from Ref. [92]). A Levene's test indicates that variance in male reproductive success is significantly higher than variance in female reproductive success (t = –3.36, p < 0.01). (N = 44 males and 48 females >42 years of age). (d) Lifetime reproductive success of the mildly polygynous Aka of the Central African Republic (re-drawn, with permission, from Ref. [87]). A Levene's test indicates that male and female variances are not significantly different (t = –1.32, n.s.). (N = 29 males and 34 females >41 years of age.) Colour code for parts (b–d): red bars, males; yellow bars, females. Abbreviations: n.s., not significant.

Unfortunately, insufficient data are available to test whether or not most relevant variables predicted from the models in Box 2, such as sex ratio or population density, explain variation between populations. Further analysis does, however, reveal that the ratio of male to female variance in RS differs significantly with mating system; monogamous societies have low ratios, whereas polygynous societies have significantly higher ratios (Figure 1a). Societies with extensive serial monogamy have ratios similar to those of polygynous societies. Across all of the monogamous societies, the ratio of variance in male to female RS is not significantly different from one (two-tailed *t*-test: *t*_5_ = 1.17, n.s.); for example, among the Pitcairn Islanders, the patterns of RS for men and women completely overlap (Figure 1b). Conversely, the data from polygynous societies is consistent with Bateman's expectation that the variance in male RS will be greater than the variance in female RS (*t*_8_ = 4.33, p = 0.003); for instance, among the Dogon of Mali, the male to female ratio of variance in RS is 4.75 (Figure 1c). However, even in polygynous societies, male and female distributions in RS can be remarkably similar; for example, among the Aka of the Central African Republic, the ratio of 1.66 is not significantly different from a ratio of 1 (Figure 1d). From these data, we conclude that the variation across populations in the ratio of male to female variance in RS is not random. The link between mating system and the ratio of male to female variance in RS is potentially mediated by those factors identified by recent theory (Box 2) to affect sexual selection (e.g. variation in mate quality, sex ratio, population density). Further data are needed to test this hypothesis.

An alternative conclusion that could be drawn from these results is that the ratio of variance in male and female RS is greatest in those populations in which male mating success varies more than female mating success (i.e. polygynous societies). However, we remain cautious about the assumption that mating success in polygynous societies is greater among males than females, for reasons outlined in the next section, and we again stress that Bateman's third principle is key to predicting the potential for sexual selection to act differently on males and females.

### Is there greater variation in MS among human males than females?

Several types of data suggest that there is greater variation in MS among males than females. For instance, in Western societies, men are more likely to re-marry than women, possibly as a result of the longer reproductive lifespan of men compared to women [29], whereas data on marriage systems around the world show that polygynous societies are far more common (83%) than monogamous (16%) or polyandrous societies (1%) [30]. Given that polygyny will lead some men to have many wives and others to have none, whereas virtually all females will have a single or a small number of successive husbands, variance in mating success is expected to be higher among human males than females in polygynous societies. There are, however, several problems with this line of reasoning.

First, in approximately half of societies formally categorized as polygynous, it is a comparatively rare event (<5%) for males to take on more than one wife [30]. As a result, even within polygynous societies, most marriages are monogamous. Moreover, because most polygynous societies are small-scale, pre-industrial societies and most large-scale societies are institutionally monogamous [30], the majority of people in (at least the contemporary) world live in institutionally monogamous societies, which might have similar variance in MS in men and women.

A second problem is that the label ‘polygyny’ does not provide any information about whether women have single or multiple partners during their lifetime. In some polygynous populations (e.g. Arsi Oromo of Ethiopia), divorce is rare [31], whereas in others (e.g. the Datoga of Tanzania), women are able to divorce and re-marry [32].

A third problem is that serially monogamous societies are typically viewed as equivalent to polygyny, with some men monopolizing more than a single female reproductive lifespan through repeated divorce and remarriage [33]. This is misleading insofar that, in serially monogamous populations such as the Dobe !Kung of Botswana [34], Ache of Paraguay [35] and Pimbwe of Tanzania [36], women and men conceive children with multiple partners. In sum, the lack of good datasets on sex differences in actual number of mating partners, and the likelihood that partner numbers vary across populations, means that it is currently difficult to make reliable generalizations about how Bateman's second principle will apply to human beings.

### What is the relationship between RS and MS in human males and females?

Although Bateman's first and second principles are often cited as if they were necessary and sufficient conditions for sex differences in the potential for sexual selection, this is not the case. The third principle, which examines the relationship between RS and MS, provides the key information from which to make predictions about how sexual selection will act on males and females to fashion sex roles. We therefore need to ask which, if any, of the relationships between RS and MS (e.g. linear, single-mate saturation, diminishing returns or intermediate optimum) apply to human beings.

For men, the relationship between RS and MS is likely to vary between populations. If men contribute heavily to the upbringing and/or inheritance of individual children, polygyny will be costly and increases in RS will not be linear [37]. In this scenario, we might predict a single-mate-saturation or diminishing-returns relationship between MS and RS for caring males. Conversely, if a male trait precipitating polygyny is easily sharable among offspring (e.g. coming from a good family, or possessing ‘good genes’) there might be no costs, and effects of mate number on RS could be linear. If polygyny is ‘wealth enhancing’ (for example, if cooperation between co-wives in polygynous households exponentially increases food production; [38]), effects of mate number on male RS could be escalating, a relationship not predicted by Arnold [8]. Finally, it is possible that RS might be severely compromised by taking additional partners, for instance if sexually transmitted diseases are prevalent.

Surprisingly, very few datasets provide demographic measures from a single population showing the effects of mate (or spouse) number on male RS. Data from the polygynous Xavante of Brazil [39] and Kipsigis of Kenya [40], and from serially monogynous modern Sweden [41], suggest that male RS is positively correlated with MS. A strong positive correlation has been found between wealth and RS in both traditional and modern populations [42]; however, this relationship does not necessarily result from an increase in mate number. For instance, a study of British men showed that wealthy men had a higher reproductive success than poorer men, but were not more likely to have had multiple spouses [42]. In addition, data from the serially monogynous Pimbwe of Tanzania show that male RS decreases with an increasing number of partners [36]. The negative relationship between MS and RS in the Pimbwe might reflect the fact that men's hunting has been rendered illegal, which potentially severely reduces men's ability to provide for the offspring of multiple partners [36].

Now consider the relationship between RS and MS for women. Evolutionary biologists have proposed several potential benefits for females that engage in polyandry, including assurance of fertilization, material gains from several mates, genetic benefits for offspring and reduced infanticide risk [21,22]. Hrdy [43] has argued that women might enhance their RS by mating with multiple partners under a range of circumstances, in particular through a gain in resources or a reduction in infanticide risk. Conversely, women might be expected to exhibit a reduction in RS by taking extra partners in populations in which the prevalence of sexually transmitted diseases is high or where there are severe socially imposed costs of taking multiple partners.

Unfortunately, very few datasets plot offspring number on spouse number for women. Among the Pimbwe, women who have three or more spouses have higher fitness than those with fewer partners [36]; however, this is likely to be an unusual pattern, possibly reflecting a situation with high and unpredictable variance in male quality over time. Other studies suggest no effects of spousal number in RS of women, as in historical Finland [44] and modern Sweden [41]; possible negative effects, as in modern Britain [42]; possible positive effects, as in modern India [45]; or positive effects of multiple fathers on child survival but not maternal RS, as in South American Indian ‘partible paternity’ populations [35,46].

To fully understand the variability in Bateman gradients across human populations, more datasets are needed that compare the effects of mate number on both male and female RS within a single population [47]. Nonetheless, the available evidence suggests that the relationship between RS and MS within each sex varies across societies.

## Implications for future research

We have argued that researchers cannot predict the shape of Bateman curves for men and women simply from Bateman's original work on fruit flies. Recent theoretical models predict that Bateman gradients are likely to vary between human populations. Unfortunately, little strong evidence is currently available to plot the shape of Bateman curves for men and women across human populations. However, we were able to collate extensive evidence on the ratio of variance in male and female RS in several different populations and to confirm that this ratio varies greatly between human populations. These data, allied with the more qualitative findings suggesting between-societal variation in MS and the RS–MS relationship, throws into question Bateman's expectation of universal human sex roles.

Recent advances in evolutionary theory (Box 2) suggest that several factors, such as sex-biased mortality, sex ratio, population density and variation in mate quality, are likely to impact on mating behaviour in human populations; these effects are potentially open to future investigation. We hope that our article will lead to future research that includes this important information. Already, some evidence exists to support these propositions; for example, a study of historical records reported stronger female choosiness in US states with a male-biased sex ratio than in states with even sex ratios [48], and strong female competition occurs in socially stratified, monogamous societies with high variation in male quality [49]. Between-population variation in sex roles should also correlate with other variables, such as population density (which might increase mate encounter rate and reduce the costs of searching for mates) and relative resource-holding power of men and women. Future research should explore both inter- and intra-population variation in male and female mating strategies based on this solid mathematical theory, rather than the idea of a single universal pattern. The insights gained from this new perspective will have important implications for how we conceive of past and current selection acting on human populations (Box 3).

## Figures and Tables

**Table 1:** Mean and variance in reproductive success (RS) of males and females in 18 populations^a^

Country	Population or ethnic group	N_m_^b^	Mean_m_	Var_m_	N_f_	Mean_f_	Var_f_	V_m_: V_f_	I_m_: I_f_	Mating system^c^	Refs
Finland	1745–1900 genealogies	125	3.4	6	138	3.5	7.6	**0.79**	0.81	Monogamy	[80]
Norway	1700–1900 genealogies	955	4.7	8.5	991	4.5	8.3	**1.02**	0.98	Monogamy	[81]
Pitcairn Island	Genealogical records	145	4.6	23.6	127	4.7	23.2	**1.02**	1.04	Monogamy	[82]
Iran	Yomut Turkmen	267	5.1	8.1	216	3.9	7.1	**1.14**	0.86	Polygyny/monandry	[83]
Sweden	1825–1896 genealogies	1201	2.1	11.5	1050	2.4	9.7	**1.18**	1.65	Monogamy	[84]
Dominica	Local population	130	4.4	14.3	124	5	11.6	**1.23**	1.40	Monogamy	[85]
Tanzania	Pimbwe	138	6.0	9	154	6.1	7.3	**1.24**	1.27	Serial monogamy	[36]
USA	General social survey	1099	2.0	2.3	1344	2.0	1.8	**1.27**	1.25	Monogamy	[86]
Central African Republic	Aka	29	6.3	8.6	34	6.2	5.2	**1.66**	1.63	Polygyny/monandry	[87]
Botswana	Dobe !Kung	35	5.1	8.6	62	4.7	4.9	**1.77**	1.61	Serial monogamy	[34]
Tanzania	Hadza	54	4.3	9.8	44	3.6	5.1	**1.93**	1.63	Polygyny/serial monandry	[88]
Venezuela	Yanomamo	279	3.7	10.1	380	3.4	4.4	**2.30**	2.11	Polygyny/monandry	[89]
Chad	Dazagada	44	8.6	15.0	33	6.4	6.5	**2.31**	1.72	Polygyny/monandry	[90]
Chad	Arabs	23	10.3	14.4	22	8.3	5.1	**2.82**	2.28	Polygyny/monandry	[90]
Brazil	Xavante	62	3.6	12.1	44	3.6	3.9	**3.10**	3.10	Polygyny/serial monandry	[39]
Kenya	Kipsigis	82	10.9	24.4	260	6.6	5.9	**4.18**	2.52	Polygyny/monandry	[91]
Paraguay	Ache	48	6.4	15.1	25	7.8	3.6	**4.22**	5.16	Serial monogamy	[35]
Mali	Dogon	44	6.1	10.7	48	3.2	2.3	**4.75**	2.47	Polygyny/serial monandry	[92]

a Most studies report lifetime RS as the number of live births, or children living to 5 or 15 years of age, for post-reproductive men and women. Where the mean RSs for males and females are not equal, the data have not been drawn from a closed population. The ratio of the opportunity for selection in males and females (I_m_: I_f_) takes into account the difference in average RS between males and females (the same pattern of results is found when this variable is used instead of V_m_: V_f_ in the analyses regarding mating systems).

b Column heading abbreviations: I_m_: I_f_,ratio of the ‘opportunity for selection’ in males and females, where I = variance in RS divided by the square of the mean RS [79]); Mean_f_, mean female lifetime RS; Mean_m_, mean male lifetime RS; N_f_, number of females; N_m_, number of males; Var_f_, variance in female RS; Var_m_, variance in male RS; V_m_: V_f_,ratio of variance in male RS to female RS.

c For non-monogamous populations, the most common mating patterns for males and females are stated separately (males/females).

## Box 1 The shape of Bateman gradients

Arnold [8] proposed four possible relationships between mating success (MS) and reproductive success (RS): linear, single-mate saturation, diminishing returns and intermediate optimum, all of which are found in both male and female animals.

The linear relationship, originally reported by Bateman [2] for male *Drosophila melanogaster*, has subsequently been reported for males in other *Drosophila* species [18] and for other male invertebrates (e.g. red-back spiders, *Latrodectus hasselti* [50]) and male vertebrates (e.g. splendid fairy wrens, *Malurus splendens* [51], and yellow-pine chipmunks, *Tamias amoenus* [52]). A linear relationship has also been found in female insects (e.g. *Drosophila simulans* [53]), birds (e.g. brown-headed cowbirds, *Molothrus ater* [54]) and mammals (e.g. prairie dogs, *Cynomys gunnisoni* [55]). In prairie dogs, litter size is positively correlated with the number of male mating partners, possibly owing to the benefits of genetic diversity among offspring [55].

In some species, there might be no increase in RS after an individual has obtained one mating partner (single-mate saturation), as reported by Bateman [2] for female *Drosophila* (but not confirmed for other *Drosophila* species [18]) and subsequently found in other invertebrate females, in which a single mating partner is apparently sufficient to sire all offspring (e.g. *L. hasselti* [50]). In some insects, males engage in suicidal copulations or effectively castrate themselves after a single mating, thereby severely limiting their ability to re-mate [56]. Among vertebrates, mating with multiple partners fails to increase RS in female bank voles (*Clethrionomys glareolus*) [57], where males provide no direct investment to females or their offspring, and in male sex-role-reversed pipefish, *Syngnathus typhle* [58], where the size of the brood pouch of the male places an upper limit on his reproductive output [59].

A diminishing returns relationship, such that progressively fewer offspring are gained with each additional mating partner, seems to occur in female *Drosophila lummei* [18] and might characterize other female insects, particularly where females accrue material benefits, such as nutritional nuptial gifts, from matings but have limited total egg production [17]. A diminishing returns relationship is also probably found in female guppies, *Poecilia reticulata* [60]. In some male insects, the costs of nuptial gifts, ejaculates or courtship might lead to a reduction in the number of successful future copulations that a male can perform [23]. The costs of sperm production and mating might also limit the benefits of extra mates for male mammals; for instance, in species in which males defend harems, male RS might plateau after harems reach a particular size (e.g. Misaki stallions, *Equus caballus* [61]), although whether males actually mate with all harem females is often unclear.

Finally, a recent meta-analysis of the insect literature supports the existence of an intermediate optimal mate number for some female insects: low MS commonly does not provide females with sufficient sperm, whereas a high MS can reduce lifespan [17] with a resulting diminution of lifetime RS. For example, female *Drosophila mojavensis* that are mated with four males have a shorter lifespan and lower lifetime RS than females that are mated with two males [62]. Courtship has been shown to reduce lifespan in male insects (e.g. Ref. [63]), which potentially means that an intermediate number of mating partners optimises male lifetime RS. In mammals, males with an intermediate harem size have been suggested to have greater RS than males with either smaller or larger harems (e.g. marmots, *Marmota flaviventris* [64]).

## Box 2 Sex roles and sexual selection theory

Recent advances in sexual selection theory have revealed that the relative choosiness and competitiveness of males and females cannot be predicted from levels of parental investment alone [25]. Here, we focus on whether males and females differ in their ‘choosiness’, defined as the proportion of potential mating partners that an individual rejects. Choosiness will increase the average quality of accepted mates, but reduce the rate at which individuals encounter acceptable partners. Kokko and Monaghan [27] investigated which factors might affect levels of choosiness in a population and established that choosiness can invade the female population if:

$$C_{F}M\sqrt{\beta }>\mu_{IF}\frac{1-p_{M}q_{M}}{p_{M}(q_{M}-1)}\text{,}$$

and the male population if:

$$C_{M}\frac{M}{\sqrt{\beta }}>\mu_{IM}\frac{1-p_{F}q_{F}}{p_{F}(q_{F}-1)}\text{,}$$

where *M* is the rate at which an individual meets receptive mates in a population of unbiased operational sex ratio (OSR), *q*_*M*_ is the increment in quality of mates gained by females who accept fraction *p*_*M*_ of males, and likewise for *q*_*F*_ and *p*_*F*_ [14,27]. The other parameters in these equations are the sex-specific costs of breeding (*C*_*M*_, *C*_*F*_), the OSR (*β*) and sex-specific mortality rates while seeking mates (*μ*_*IM*_*, μ*_*IF*_). Thus, whether a sex is choosy depends on: (i) the sex-specific mortality cost of breeding (*C*_*F*_*, C*_*M*_), where high costs favour choosiness (this is well captured by the concept of *parental investment* [3]); (ii) rate of encountering mates ($M\sqrt{\beta }$, $M/\sqrt{\beta }$), where a high rate of encounter engenders choosiness. High contact rates (large *M*) favour choosiness in both sexes, whereas a biased OSR (*β*) favours greater choosiness in the rarer sex; (iii) sex-specific mortality while seeking mates (*μ*_*IF*_*, μ*_*IM*_), where a low cost of mate searching favours choosiness; and (iv) variation in mate quality, which depends upon the degree of improvement in mate quality, *q*, and rate of encountering high quality mates, *p*. If mate quality is highly variable, substantial improvements in quality can be achieved without greatly reducing the mating rate, generating the large *qp* that favours choosiness.

Kokko and Monaghan [27] found that competitiveness will also be affected by sex-specific mortality costs and mortality rates, and the OSR. These factors are expected to vary between species and across populations of the same species. Predicting which sex invests most heavily in parental care is equally complex and multiply determined [25]. For instance, male care can evolve if the gains derived by increasing offspring fitness outweigh the lost mating opportunities while caring, and the latter are diminished where there is a male-biased adult sex ratio [25]. In summary, current sexual selection theory indicates that variables such as choosiness and competitiveness are influenced by multiple factors and provides little support for the view that a single sex-role stereotype will apply universally to all human populations.

## Box 3 Implications for evolutionary psychology

Evolutionary psychologists assume that human behaviour is guided by evolved psychological mechanisms that were selected to solve problems commonly encountered by our hominin ancestors [65]. Bateman's principles have formed the cornerstone of a large field of evolutionary psychology dedicated to understanding sex differences in human traits, such as mate-choice preferences, sexual jealousy and aggression (e.g. see Refs [66,67,68]). Human beings are commonly characterized as monogamous with polygamous tendencies, such that men are predisposed to commit paternal investment but seek out additional mating partners where possible, whereas women are predisposed to seek monogamous relationships but occasionally engage in extra-pair matings with high-quality males [69]. Evolutionary psychologists do consider within-sex variation in mating behaviour, commonly in terms of condition-dependent strategies; human beings are assumed to have inherited a diverse repertoire of sexual strategies, such that developmental, social and ecological conditions can evoke particular strategies that will have been adaptive in our evolutionary past [70,71].

Our finding that variance in male RS exceeds variance in female RS (when all datasets in Table 1 are considered together) could be seen as being consistent with the evolutionary psychology perspective, whereas the between-population variation in the ratio of male to female RS could be viewed as diverse sexual strategies evoked from universal, evolved psychological mechanisms. However, we envisage several challenges to this viewpoint. First, evidence for sex differences in variation in RS alone does not enable us to make generalizations about sex roles because numerous variables will influence the shapes of the Bateman gradients for men and women, and variance does not necessarily imply selection (Box 2). Second, although evolutionary psychology has traditionally emphasized ‘innate’, context-specific structure in the mind, the more flexible and variable the exhibited behaviour, the less explanatory power can be attributed to such structure.

Third, although we anticipate that ecological and cultural variation are the primary sources of the observed differences between human societies, we are open to the possibility that this variation is supplemented by variation in evolved predispositions across populations. The long-held assumption within evolutionary psychology that the human brain consists of mental adaptations that are products of resistant-to-change gene complexes must be re-examined [72]. Extensive recent data reveal that many hundreds of human genes (up to 10% of the genome, [73]) have been favoured by recent selective sweeps during the past 50 000 or so years [74], including many genes expressed in the brain [75]. Such studies indicate that recent selective sweeps have frequently been population-specific [74,75]; for instance, genetic variation is associated with cross-cultural diversity in the use of tonal languages [76]. Gene-culture co-evolutionary models reinforce the view that variation in learning mating behaviour and cultural belief systems can generate divergent selection on human genes [77,78]. Confirmation of relevant genetic variation would further reduce the likelihood that human mating strategies conform to a single universal pattern.

## Glossary

Bateman's first principle: states that males show greater variance in number of offspring than females.
Bateman's second principle: states that males show greater variance in number of sexual partners than females.
Bateman's third principle: states that there is a stronger relationship between reproductive success and mating success among males than females.
Choosiness: the proportion of potential mating partners that an individual rejects.
Competitiveness: the intensity with which an individual competes with members of the same sex.
Monandry: females usually mate with only one partner during their lifetime.
Monogamy: both males and females usually mate with only one partner during their lifetime.
Operational sex ratio (OSR): the ratio of reproductively available males to females.
Polygyny: males have multiple mates concurrently.
Serial monandry: females usually sequentially mate with more than one partner during their lifetime.
Serial monogamy: both males and females sequentially mate with several partners during their lifetime.
Sex roles: the set of behaviour patterns that is expected to be shown by males and females when competing for or choosing mates (i.e. relative competitiveness and choosiness of males and females).

## References

[1] Darwin C. (1871). The Descent of Man and Selection in Relation to Sex.

[2] Bateman A.J. (1948). Intra-sexual selection in *Drosophila*. Heredity.

[3] Trivers R.L., Campbell B. (1972). Parental investment and sexual selection. Sexual Selection and the Descent of Man, 1871–1971.

[4] Emlen S.T., Oring L.W. (1977). Ecology, sexual selection, and the evolution of mating systems. Science.

[5] Clutton-Brock T.H., Parker G.A. (1992). Potential reproductive rates and the operation of sexual selection. Q. Rev. Biol..

[6] Clutton-Brock T.H. (1988). Reproductive Success: Studies of Individual Variation in Contrasting Breeding Systems.

[7] Trivers R. (1985). Social Evolution.

[8] Arnold S.J. (1994). Bateman's principles and the measurement of sexual selection in plants and animals. Am. Nat..

[9] Snyder B.F., Gowaty P.A. (2007). A reappraisal of Bateman's classic study of intrasexual selection. Evolution Int. J. Org. Evolution.

[10] Sutherland W.J. (1985). Chance can produce a sex difference in variance in mating success and explain Bateman's data. Anim. Behav..

[11] Gowaty P.A., Hubbell S.P. (2005). Chance, time allocation, and the evolution of adaptively flexible sex role behavior. Integr. Comp. Biol..

[12] Arnold S.J., Duvall D. (1994). Animal mating systems: a synthesis based on selection theory. Am. Nat..

[13] Andersson M., Iwasa Y. (1996). Sexual selection. Trends Ecol. Evol..

[14] Kokko H., Johnstone R.A. (2002). Why is mutual mate choice not the norm? Operational sex ratios, sex roles and the evolution of sexually dimorphic and monomorphic signalling.. Philos. Trans. R. Soc. Lond., B.

[15] Forsgren E. (2004). Unusually dynamic sex roles in a fish. Nature.

[16] Ridley M. (1988). Mating frequency and fecundity in insects. Biol. Rev. Camb. Philos. Soc..

[17] Arnqvist G., Nilsson T. (2000). The evolution of polyandry: multiple mating and female fitness in insects. Anim. Behav..

[18] Bjork A., Pitnick S. (2006). Intensity of sexual selection along the anisogamy-isogamy continuum. Nature.

[19] Gowaty P.A. (2003). Indiscriminate females and choosy males: within and between-species variation in *Drosophila*. Evolution Int. J. Org. Evolution.

[20] Hrdy S.B. (1981). The Woman That Never Evolved.

[21] Jennions M.D., Petrie M. (2000). Why do females mate multiply? A review of the genetic benefits. Biol. Rev. Camb. Philos. Soc..

[22] Wolff J.O., Macdonald D.W. (2004). Promiscuous females protect their offspring. Trends Ecol. Evol..

[23] Bonduriansky R. (2001). The evolution of male mate choice in insects: a synthesis of ideas and evidence. Biol. Rev. Camb. Philos. Soc..

[24] Wedell N. (2002). Sperm competition, male prudence and sperm-limited females. Trends Ecol. Evol..

[25] Kokko H., Jennions M.D. (2008). Parental investment, sexual selection and sex ratios. J. Evol. Biol..

[26] Johnstone R.A. (1996). Mutual mate choice and sex differences in choosiness. Evolution Int. J. Org. Evolution.

[27] Kokko H., Monaghan P. (2001). Predicting the direction of sexual selection. Ecol. Lett..

[28] Low B.S. (1988). Measures of polygyny in humans. Curr. Anthropol..

[29] Buckle L. (1996). Marriage as a reproductive contract: patterns of marriage, divorce, and remarriage. Ethol. Sociobiol..

[30] Murdock G.P. (1967). Ethnographic Atlas.

[31] Gibson M.A., Mace R. (2007). Polygyny, reproductive success and child health in rural Ethiopia: why marry a married man?. J. Biosoc. Sci..

[32] Borgerhoff Mulder M. (1992). Demography of pastoralists: preliminary data on the Datoga of Tanzania. Hum. Ecol..

[33] Starks P.T., Blackie C.A. (2000). The relationship between serial monogamy and rape in the United States (1960–1995). Proc. R. Soc. Lond. B. Biol. Sci..

[34] Howell N. (1979). Demography of the Dobe! Kung.

[35] Hill K., Hurtado A.M. (1996). Ache Life History: The Ecology and Demography of a Foraging People.

[36] Borgerhoff Mulder, M. (2009) Serial monogamy as polygyny or polyandry? Marriage in the Tanzanian Pimbwe. *Hum. Nat.* 20 (in press) 10.1007/s12110-009-9060-x PMC5486523 25526955

[37] Geary D.C. (2000). Evolution and proximate expression of human parental investment. Psychol. Bull..

[38] White D.R. (1988). Rethinking polygyny: co-wives, codes, and cultural systems. Curr. Anthropol..

[39] Salzano F.M. (1967). Further studies on the Xavante Indians. I. Demographic data on two additional villages: genetic structure of the tribe. Am. J. Hum. Genet..

[40] Borgerhoff Mulder M. (1987). On cultural and reproductive success: Kipsigis evidence. Am. Anthropol..

[41] Forsberg A.J.L., Tullberg B.S. (1995). The relationship between cumulative number of cohabiting partners and number of children for men and women in modern Sweden. Ethol. Sociobiol..

[42] Nettle D., Pollet T.V. (2008). Natural selection on male wealth in humans. Am. Nat..

[43] Hrdy S.B. (1997). Raising Darwin's consciousness: female sexuality and the prehominid origins of patriarchy. Hum. Nat..

[44] Käär P. (1998). Sexual conflict and remarriage in preindustrial human populations: causes and fitness consequences. Evol. Hum. Behav..

[45] Leonetti D.L. (2007). In-law conflict: women's reproductive lives and the roles of their mothers and husbands among the matrilineal Khasi. Curr. Anthropol..

[46] Beckerman S., Valentine P. (2002). Cultures of Multiple Fathers.

[47] Borgerhoff Mulder M. (2004). Are men and women really so different?. Trends Ecol. Evol..

[48] Pollet T.V., Nettle D. (2008). Driving a hard bargain: sex ratio and male marriage success in a historical US population. Biol. Lett..

[49] Gaulin S.J.C., Boster J.S. (1990). Dowry as female competition. Am. Anthropol..

[50] Andrade M.C.B., Kasumovic M.M. (2005). Terminal investment strategies and male mate choice: extreme tests of Bateman. Integr. Comp. Biol..

[51] Webster M.S. (2007). Promiscuity drives sexual selection in a socially monogamous bird. Evolution Int. J. Org. Evolution.

[52] Schulte-Hostedde A.I. (2004). Sexual selection and mating patterns in a mammal with female-biased sexual size dimorphism. Behav. Ecol..

[53] Taylor M.L. (2008). Multiple mating increases female fitness in *Drosophila simulans*. Anim. Behav..

[54] Woolfenden B.E. (2002). High opportunity for sexual selection in both sexes of an obligate brood parasitic bird, the brown-headed cowbird (*Molothrus ater*). Behav. Ecol. Sociobiol..

[55] Hoogland J.L. (1998). Why do female Gunnison's prairie dogs copulate with more than one male?. Anim. Behav..

[56] Miller J.A. (2007). Repeated evolution of male sacrifice behavior in spiders correlated with genital mutilation. Evolution Int. J. Org. Evolution.

[57] Mills S.C. (2007). Quantitative measure of sexual selection with respect to the operational sex ratio: a comparison of selection indices. Proc. R. Soc. Lond. B. Biol. Sci..

[58] Jones A.G. (2000). The Bateman gradient and the cause of sexual selection in a sex-role-reversed pipefish. Proc. R. Soc. Lond. B. Biol. Sci..

[59] Berglund A. (1989). Reproductive success of females limited by males in two pipefish species. Am. Nat..

[60] Becher S.A., Magurran A.E. (2004). Multiple mating and reproductive skew in Trinidadian guppies. Proc. R. Soc. Lond. B. Biol. Sci..

[61] Kaseda Y., Khalil A.M. (1996). Harem size and reproductive success of stallions in Misaki feral horses. Appl. Anim. Behav. Sci..

[62] Etges W.J., Heed W.B. (1992). Remating effects on the genetic structure of female life histories in populations of *Drosophila mojavensis*. Heredity.

[63] Mappes J. (1996). Viability costs of condition-dependent sexual male display in a drumming wolf spider. Proc. R. Soc. Lond. B. Biol. Sci..

[64] Downhower J.F., Armitage K.B. (1971). The yellow-bellied marmot and the evolution of polygamy. Am. Nat..

[65] Tooby J., Cosmides L. (1990). The past explains the present: emotional adaptations and the structure of ancestral environments. Ethol. Sociobiol..

[66] Symons D. (1979). The Evolution of Human Sexuality.

[67] Daly M., Wilson M. (1983). Sex, Evolution and Behavior.

[68] Buss D.M. (1994). The Evolution of Desire: Strategies of Human Mating.

[69] Buss D.M. (2008). Evolutionary Psychology: The New Science of the Mind.

[70] Gangestad S.W., Simpson J.A. (2000). The evolution of human mating: trade-offs and strategic pluralism. Behav. Brain Sci..

[71] Tooby J., Carruthers P. (2005). Resolving the debate on innate ideas: learnability constraints and the evolved interpenetration of motivational and conceptual functions. The Innate Mind: Structure and Content.

[72] Laland K.N., Brown G.R. (2002). Sense and Nonsense: Evolutionary Perspectives on Human Behaviour.

[73] Williamson S.H. (2007). Localizing recent adaptive evolution in the human genome. PLoS Genet..

[74] Sabeti P.C. (2006). Positive natural selection in the human lineage. Science.

[75] Wang E.T. (2006). Global landscape of recent inferred Darwinian selection for *Homo sapiens*. Proc. Natl. Acad. Sci. U. S. A..

[76] Dediu D., Ladd D.R. (2007). Linguistic tone is related to the population frequency of the adaptive haplogroups of two brain size genes, *ASPM and Microcephalin*. Proc. Natl. Acad. Sci. U. S. A..

[77] Boyd R., Richerson P.J. (1985). Culture and the Evolutionary Process.

[78] Mesoudi A., Laland K.N. (2007). Culturally transmitted paternity beliefs and the evolution of human mating behaviour. Proc. R. Soc. Lond. B. Biol. Sci..

[79] Shuster S.M., Wade M.J. (2003). Mating Systems and Strategies.

[80] Pettay J.E. (2005). Heritability and genetic constraints of life-history trait evolution in preindustrial humans. Proc. Natl. Acad. Sci. U. S. A..

[81] Røskaft E. (1992). Reproductive success in relation to resource-access and parental age in a small Norwegian farming parish during the period 1700–1900. Ethol. Sociobiol..

[82] Brown D.E., Hotra D., Betzig L. (1988). Are prescriptively monogamous societies effectively monogamous?. Human Reproductive Behaviour. A Darwinian Perspective.

[83] Irons W., Cronk L. (2000). Why do the Yomut raise more sons than daughters?. Adaptation and Human Behavior: An Anthropological Perspective.

[84] Low B.S. (1991). Reproductive life in nineteenth century Sweden: an evolutionary perspective on demographic phenomena. Ethol. Sociobiol..

[85] Quinlan R.J., Flinn M.V. (2005). Kinship, sex, and fitness in a Caribbean community. Hum. Nat..

[86] Weeden J. (2006). Do high-status people really have fewer children? Education, income, and fertility in the contemporary U.S.. Hum. Nat..

[87] Hewlett B.S., Betzig L. (1988). Sexual selection and paternal investment among Aka pygmies. Human Reproductive Behaviour. A Darwinian Perspective.

[88] Marlowe F. (2000). The patriarch hypothesis: an alternative explanation of menopause. Hum. Nat..

[89] Chagnon N., Chagnon B.N.A., Irons W. (1979). Is reproductive success equal in egalitarian societies?. Evolutionary Biology and Human Social Behavior.

[90] Fazzio I. (2008). Parental Investment among Arab and Dazagada Herding Societies of West Chad.

[91] Borgerhoff Mulder M., Clutton-Brock T.H. (1988). Reproductive success in three Kipsigis cohorts. Reproductive Success: Studies of Individual Variation in Contrasting Breeding Systems.

[92] Strassmann B.I., Reichard U.H., Boesch C. (2003). Social monogamy in a human society: marriage and reproductive success among the Dogon. Monogamy: Mating Strategies and Partnerships in Birds, Humans and Other Mammals.

